# Perceived Digital Well-Being Scale in the United States and United Kingdom: Psychometric Validation Study

**DOI:** 10.2196/78334

**Published:** 2025-10-30

**Authors:** Germano Vera Cruz, Magdalena Liberacka-Dwojak, Monika Wiłkość-Dębczyńska, Merve Aktaş Terzioğlu, Todd Farchione, Tania Lecomte, Sandy Ingram, Riaz Khan, Yasser Khazaal

**Affiliations:** 1 Department of Psychology, Université de Picardie Jules Verne, Amiens, France; 2 Department of Health Psychology, Faculty of Psychology, Kazimierz Wielki University, Bydgoszcz, Poland; 3 Department of Child and Adolescent Psychiatry, Faculty of Medicine, Pamukkale University, Denizli, Turkey; 4 Department of Psychology, University of Boston, Boston, MA, United States; 5 Department of Psychology, Université de Montréal, Montreal, Canada; 6 Institute for Intelligent and Secure Systems, School of Engineering and Architecture, University of Applied Sciences of Western Switzerland, Fribourg, Switzerland; 7 Department of Psychiatry, Frontier Medical College, Bahria University, Islamabad, Pakistan; 8 Department of Psychiatry, Addiction Medicine, Lausanne University Hospital and Lausanne University, Rue du Bugnon 23, Lausanne, 1011, Switzerland, 41 766792018

**Keywords:** digital connectivity, Perceived Digital Well-Being Scale, smartphone, social media, digital well-being, digital flourishing, digital stress

## Abstract

**Background:**

Digital well-being encourages balanced mobile use. The Perceived Digital Well-Being in Adolescence Scale measures this in adolescents but has been validated only in Slovenia, raising questions about its relevance for other age groups and cultural contexts.

**Objective:**

This study had three primary objectives: (1) confirm the 3-factor structure of an English version of the Perceived Digital Well-Being in Adolescence Scale, renamed the Perceived Digital Well-Being Scale (PDWS), in samples of young adults from the United States and the United Kingdom; (2) examine the associations between PDWS dimensions and participants’ sociodemographic characteristics; and (3) explore the relationships between PDWS scores and patterns of smartphone use.

**Methods:**

A total of 1854 young adults from the United States and the United Kingdom (ages 18‐25 years; mean 22.4, SD 2.1; 892, 48.1% female, 872, 47.0% male, 90, 4.9% nonbinary) participated in an online survey including the PDWS, digital flourishing, and digital stress measures. Data were analyzed using descriptive statistics, confirmatory factor analysis, correlations, *t* tests, chi-squared tests, and moderation-mediation analysis.

**Results:**

Smartphone screen time and smartphone time for nonessential activities were statistically higher in the US sample than in the UK sample (mean 6.95 vs mean 6.13; *t*_1852_=4.97; *P*<.001; *d*=0.27 and mean 3.62 vs mean 3.29; *t*_1852_=5.57; *P*<.001; *d*=0.25, respectively). The digital well-being total score was statistically higher among US participants when compared with the UK counterparts (mean 3.49 vs mean 3.38; *t*_1852_=3.33; *P*<.001; *d*=0.15). Male participants were significantly more represented among the group with higher PDWB scores (*χ*^2^_136_=478.45; *P*<.001). Confirmatory factor analysis supported the adequacy of the 3-factor model (emotional, social, and cognitive), indicating strong model fit (critical indices ≥0.90). Evidence of convergent validity was established through significant associations between PDWS scores and measures of digital flourishing and digital stress (most correlation coefficients being significant at *P*<.001). Measurement invariance testing confirmed the scale’s equivalence across US and UK samples (*χ*^2^_246_ change=17.90; *P*=.21); however, strict invariance across gender (male vs female) was not supported (*χ*^2^_246_ change=200.91; *P*<.001). Gender, sexual orientation, relationship status, ethnicity, socioeconomic status, and education level significantly influenced PDWS scores. Gender and socioeconomic status also moderated the relationship between smartphone use or screen time and PDWS scores (*b*=0.048; *P*=.03 and *b*=0.020; *P*=.03, respectively), indicating that these factors affect how smartphone usage relates to psychological distress or well-being in different demographic groups.

**Conclusions:**

The PDWS showed good psychometric properties in the US and UK samples. The scale offers a promising tool for identifying individuals at risk of adverse outcomes associated with digital connectivity.

## Introduction

### Background

Smartphone use has become widespread in modern societies, particularly among young adults. This age group differs from older ones by having higher involvement in smartphone use and a greater propensity to use the devices for social interactions, dating, mental health, and well-being services [1-7]. Difficulties related to mastering smartphone use and specific digital services, such as social media, have been reported in numerous studies from multiple countries [8,9]. Such difficulties may, for some individuals, involve substantial time devoted to these platforms, which is not necessarily problematic in itself but can become so under certain conditions. These challenges are often conceptualized through 2 distinct frameworks. The first is digital stress [10], which refers to the psychological strain arising from constant connectivity, social expectations, approval anxiety, fear of missing out, and online vigilance. The second is compulsive smartphone and social media use [8,11,12], which emphasizes a behavioral pattern in which use becomes difficult to control and begins interfering with daily functioning and well-being.

In contrast, increasing attention has been given to the concept of digital flourishing [13,14], which highlights the positive potential of digital technologies. Digital flourishing refers to how online experiences can promote connectedness, self-expression, and well-being, emphasizing functional and social benefits rather than risks. Smartphones and digital platforms can support meaningful relationships, civic participation, and access to educational, professional, and health resources. They also create opportunities for authentic self-disclosure, identity development, and peer support, fostering resilience and psychological growth [15]. Taken together, these findings resonate with social cognitive theories [16], which emphasize the reciprocal interaction between individual factors, behaviors, and social contexts, suggesting that smartphone use is not inherently beneficial or harmful but acquires meaning and impact through the ways it is motivated, enacted, and socially reinforced. As these contrasting perspectives suggest, shifts in how digital technologies are experienced may become central to understanding broader notions of well-being and, more specifically, digital well-being [17].

Well-being is a multifaceted construct that transcends the mere absence of disease [18]. It encompasses both emotional and cognitive dimensions of subjective experience, serving as both a contributor to and an outcome of robust physical and psychological health [18]. This state of well-being includes feelings of safety, positive affect, life satisfaction, active engagement, personal fulfillment, and the presence of meaningful social connections [19-24].

Digital well-being, on the other hand, refers to a person’s individual perception of maintaining a healthy balance between the advantages and disadvantages of being connected through mobile technology [17,25]. It encompasses both emotional and cognitive evaluations of how digital connectivity fits into everyday routines [25]. People experience digital well-being when they derive maximum enjoyment and practical benefits from technology use while experiencing minimal disruptions, loss of control, or negative impacts on their overall functioning [25]. According to Vanden Abeele [25], enhancing digital well-being involves “optimizing the ambivalence by carefully adjusting our connectivity so that it provides us with controlled pleasure and maximally supports us to achieve our goals, while causing a minimal degree of functional impairment and loss of control” (p 937).

### Development of Digital Well-Being Assessment Tools

In alignment with Vanden Abeele’s [25] conceptual model of digital well-being, 2 self-report instruments have recently been developed to operationalize this construct. The first, the Perceived Digital Well-Being in Adolescence (PDWBA) Scale, was created by Rosič et al [17] following scale development guidelines outlined by Carpenter [26]. A multidisciplinary team—PhD candidates, methodologists, digital media effects experts, and researchers specializing in adolescent development—designed 17 items to capture adolescents’ subjective perceptions of the benefits and drawbacks of smartphone use. The PDWBA assesses opposing experiences of smartphone use across 3 key life domains: emotional, social, and cognitive. This scale was validated using a sample of 1040 Slovenian adolescents [17].

The second instrument*,* the Digital Well-Being Questionnaire (DWQ), was developed by Priyanka [27] to assess digital well-being through a multiphase, mixed methods approach. The initial qualitative phase involved in-depth interviews with subject matter experts, academics, and end users to explore conceptualizations of digital well-being and identify its key dimensions. These insights informed item generation, which was then evaluated for validity by an expert panel. A subsequent quantitative study confirmed the reliability and validity of the 13-item scale version. The DWQ identifies 4 dimensions of digital well-being: physical care (eg, reduced distraction from screen use), emotional resilience (eg, minimal frustration in digital contexts), agency (eg, control over screen habits), and communion (eg, connection with others through technology). These dimensions reflect both cognitive and affective evaluations of digital engagement. Cognitive appraisal pertains to comparing one’s current and ideal screen use, where smaller discrepancies are linked to more positive experiences. In contrast, affective appraisal pertains to emotional responses, such as whether screen engagement is perceived as pleasant or unpleasant.

Together, these findings underscore the multidimensional nature of digital well-being and provide a validated framework for its assessment.

### Digital Well-Being, Sociodemographic, Individual, and Behavioral Variables

Two studies conducted by Rosič et al [17,28] have identified several sociodemographic and individual factors associated with digital well-being, as measured by the PDWBA. First, gender differences were evident, with girls reporting lower levels of digital well-being than boys. Second, age-related trends were observed, indicating that older adolescents tended to score higher across all PDWBA dimensions. Third, educational background was influential: adolescents enrolled in vocational education reported significantly lower digital well-being in the social domain than their peers in general education. Fourth, a positive between-person association was found between emotional digital well-being and self-esteem, although similar associations were not observed for the social or cognitive dimensions. Finally, smartphone screen time showed a modest negative association with cognitive aspects of digital well-being. Similarly, Priyanka’s [27] study using DWQ found that higher levels of digital literacy, social capital, self-efficacy, and subjective well-being positively predicted digital well-being. Conversely, digital addiction emerged as a significant negative predictor. Collectively, these findings underscore the complex and multifaceted nature of digital well-being, shaped by both individual traits and contextual factors.

### PDBWA and DWQ: Main Differences

The PDBWA [17] comprises items designed to capture opposing ends of the digital well-being continuum, with most items structured in a “more” versus “less” format (eg, “Because of my smartphone use, I feel more excluded from my friends, or I feel closer to my friends”). This instrument conceptualizes smartphones as meta-media—platforms that provide access to a variety of applications relevant to users, including social media and mass media content. It asks participants to provide general evaluations of the perceived benefits and drawbacks of smartphone use. Notably, the PDBWA was specifically developed for adolescents.

In contrast, the DWQ [27] consists of items reflecting digital consumption experiences, framed as affirmative statements to which respondents indicate their level of agreement or disagreement (eg, “I can manage any emotional challenges caused by my screen use”). Unlike the PDBWA, the DWQ does not conceptualize smartphones as meta-media. Instead, it addresses broader aspects of digital engagement, including screen time across various digital devices, social media use, and participation in the digital world or community. Although originally validated using a sample composed of both young and older adults, the DWQ is considered suitable for use with adolescents as well.

Despite their conceptual differences, both instruments capture the benefits and drawbacks of digital connectivity and have demonstrated strong psychometric properties. For both scales, however, the authors [17,27] recommended further validation across different cultural contexts and demographic groups.

### This Study’s Purpose

In contemporary societies, individuals—particularly those aged 16 to 25 years—are increasingly confronted with complex decisions regarding when, where, how, and to what extent they engage with smartphone-based services, such as social media, online gaming, cybersexual platforms, and internet-enabled shopping. While the use of smartphones and social media has been associated with various mental health challenges, functional impairments, and psychological issues, it has also been linked to psychosocial and economic benefits. The concept of digital well-being, as previously defined, offers a valuable framework for raising awareness of the need to strike a healthy balance between the advantages and disadvantages of mobile connectivity. This conceptualization holds particular promise for informing the development of targeted interventions when this balance becomes disrupted or skewed toward negative outcomes.

However, digital well-being has become a critical concept for understanding how individuals navigate the opportunities and challenges of constant mobile connectivity [29]. While earlier research often emphasized screen time [30], compulsive smartphone use [31], and social media use [32], more recent work stresses the importance of capturing both positive and negative subjective experiences of social media use [33] across social, cognitive, and emotional domains [25,34]. The development of the PDWBA Scale marked an important step in this direction, but its validation was limited to Slovenian adolescents [17], raising questions about its broader applicability. Extending this measure to young adults, testing it across diverse cultural contexts is therefore essential. This focus on young adults is particularly important given their high involvement in smartphone and social media use [9,35], as well as the developmental phase they are navigating [36,37], where identity formation, social connectedness, and self-regulation are especially salient. By validating the English version of the scale among large samples in the United States and the United Kingdom, this study provides a reliable tool for assessing digital well-being beyond adolescence. A further contribution lies in testing measurement invariance across gender, which aims to assess areas of overlap as well as potential limits to strict comparability between male and female respondents.

In response to these limitations, this study had three primary objectives: (1) examine the construct validity—specifically, the factorial structure, scale reliability, and internal consistency—of the English-language version of the PDWBA, adapted for both adolescents and adults and hereafter referred to as the Perceived Digital Well-being Scale (PDWS), within a large sample of young adults from the United States and the United Kingdom; (2) evaluate the scale’s convergent validity by comparing it with 2 related instruments: the Digital Flourishing Scale (DFS [13,14]) and the multidimensional Digital Stress Scale (DSS [10]); and (3) explore associations between the PDWS dimensions and participants’ sociodemographic characteristics as well as patterns of smartphone use.

The PDWS used in this study is an adapted version of the original instrument, revised to be suitable for both adolescent and adult populations. This adaptation was informed by the recommendations of the original authors, as outlined in the “limitations” and “future research” sections of their publication [17]. Details regarding the adaptation process are provided in the “Methods” section below.

It is important to note that while this study focused on validating the adult version of the PDWBA in populations from the United States and the United Kingdom, a separate study aimed at validating the DWQ in North American and European populations is currently underway.

## Methods

### Participants

A total of 1854 young adults aged 18 to 25 (mean 22.4, SD 2.1) years participated in the study by completing an online questionnaire. Of these, 934 (50.5%) participants resided in the United States and 920 (49.4%) in the United Kingdom. In terms of gender identity, 892 (48.1%) identified as female, 872 (47.0%) as male, and 90 (4.9%) as nonbinary.

Given the exploratory nature of the study, a formal power analysis was not conducted. Instead, sample size determination was guided by the commonly recommended heuristic of recruiting a minimum of 10 participants per survey item [38]. The 3 primary scales used in this study comprised 65 items in total. Thus, the study had to include a minimum of 650 participants per country. The final sample sizes in both countries exceeded this threshold.

### Recruitment and Sampling

Participants were recruited anonymously through Prolific [39], a private company specializing in research participant recruitment and data collection. Prolific offers quality assurance measures and maintains a large, prescreened pool of individuals who consent to participate in online scientific research. Compared to similar platforms, Prolific has been noted for its exclusive focus on academic research and for providing access to a more ethnically and geographically diverse participant base, representative in terms of age and gender [40]. The screening criteria for study inclusion were: (1) aged 18‐25 years, and (2) daily smartphone and social media use. To ensure a gender-sensitive sampling approach, based on a target sample of 2160 (85%) participants, a quota system was chosen to obtain 42% males, 42% females, and 16% nonbinary participants. However, in the end, only 1854 participants completed the survey.

### Data Collection Material

Data were collected via an online survey that included the following components:

1. Sociodemographic questions (7 variables): participants reported age, gender (male, female, or nonbinary), relationship status (not in relationship, in a relationship but not married, or in relationship and married), level of education (measured in years of schooling), and perceived socioeconomic status (SES; low, intermediate, or high).
2. Smartphone and social media behavior: participants reported their smartphone screen time (hours per day on a typical day using the smartphone for nonessential activities during the last 3 months) and their first and second most frequently used apps.
3. The PDWS: a 17-item self-report measure covering 3 domains: emotional domain (7 items; an example item is “Because of my smartphone use, I do fewer fun things or I do more fun things.”); social domain (6 items; an example item is “Because of my smartphone use, I feel more excluded from my friends or I feel closer to my friends.”); and cognitive domain (4 items; an example item is “Because of my smartphone use, I feel I am a failure or I feel satisfied with myself.”). The response scale ranges from 1 (left pole) to 5 (right pole), with higher scores indicating higher digital well-being. In previous studies, the PDWS presented high statistical indices of reliability (Cronbach α=0.70) and structural validity (eg, comparative fit index [CFI] and Tucker-Lewis index [TLI] >0.90; root mean square error of approximation [RMSEA] <0.08) [17,28].

As stated in the “Introduction” section, originally, this digital well-being scale [17] was conceived for an adolescent population. We adapted it for young adults by adding contextual words on 3 items from the cognitive dimension. For instance, the item “Because of my smartphone use, I do less of my daily tasks (eg, schoolwork) or I do more of my daily tasks (eg, schoolwork)” became “Because of my smartphone use, I do less of my daily tasks (eg, schoolwork, university work, and professional work)” or I do more of my daily tasks (eg, schoolwork, university work, and professional work).” We similarly adapted items referring to schoolwork by adding the terms “university” and “work.” In this study, the PDWS’ internal consistency (Cronbach α) for each of the above dimensions was 0.86, 0.87, and 0.83, respectively.

The DFS [13,14] is a 25-item self-report measure assessing digital flourishing among 5 dimensions: connectedness (combination of social connection and social support; 5 items, eg, “I feel part of a community when I interact with others online”); self-control (5 items; eg, “When I interact with others about politics online, I know how to have a civil discussion.”); civil participation (5 items; eg,“Comparing myself to others online motivates me to accomplish my goals”); positive social comparison (5 items; eg, “I allow my social network to see who I really am”); and authentic self-disclosure (5 items; eg, “For the most part, I feel in control of how much time I spend interacting with others online”). A 7-point Likert scale (from 1=strongly disagree to 7=strongly agree) was applied; higher scores indicate higher flourishing. In this study, the scale’s internal consistency for each of the above dimensions was: 0.91, 0.82, 0.86, 0.96, 0.84, respectively. Previous studies conducted with North American and European samples [13,14,41] have demonstrated that the DFS possesses high reliability (Cronbach α>0.70) and strong structural validity (eg, CFI and TLI >0.90; RMSEA <.08).

The multidimensional DSS [10] is a 23-item self-report measure used to assess digital stress among 5 dimensions: connection overload (6 items; eg, “I feel overwhelmed with the flow of messages or notifications on my phone”); availability stress (4 items; eg, “My friends expect me to be constantly available online”); approval anxiety (5 items; eg, “I am nervous about how people will respond to my posts and photos”); fear of missing out (4 items; eg, “I fear my friends are having more rewarding experiences than me”); and online vigilance (4 items; eg, “I must have my phone with me to know what is going on”). A 7-point Likert scale (from 1=strongly disagree to 7=strongly agree) was applied; higher scores indicate higher digital stress. The scale’s internal consistency for each of the above dimensions was 0.86, 0.87, 0.88, 0.86, and 0.85, respectively. Previous studies [42-44] have demonstrated that the multidimensional DSS exhibits strong psychometric properties. Specifically, research conducted with North American, European, and Asian samples has shown high internal consistency, with Cronbach α values exceeding 0.70 across subscales. In addition, structural validity has been supported through confirmatory factor analyses, with model fit indices generally meeting acceptable thresholds (eg, CFI and TLI >.90; RMSEA <.08). These findings indicate that the DSS is a reliable and valid instrument for assessing various dimensions of digital stress in diverse populations.

### Statistical Analyses

First, we conducted descriptive analyses (range, mean, SD, skewness, and kurtosis).

Second, to assess the appropriateness of the data for factorial analyses (assumptions of adequacy and sphericity), we applied the Kaiser–Meyer–Olkin test and the Bartlett test of sphericity, respectively.

Third, we conducted confirmatory factor analysis (CFA) using maximum likelihood estimation to test the dimensionality and construct validity of the PDWS. The maximum likelihood estimation has proven to be effective even for categorical variables, such as those obtained from Likert-type scales, especially in relatively large samples [45,46]. To evaluate model fit, we used indices most commonly recommended by statisticians [47], including the goodness-of-fit index (GFI), normed fit index (NFI), relative fit index (RFI), incremental fit index (IFI), TLI, CFI, RMSEA, and standardized root mean square residual (SRMR). According to Goretzko et al [47] and Hu and Bentler [48], cutoffs of GFI, NFI, RFI, IFI, TLI, and CFI ≥0.95 and ≥0.90 indicate excellent and good fit, respectively; SRMR ≤0.08 and RMSEA ≤0.08 indicate good model fit. In addition, we conducted a test of factorial structure invariance between the sample of 2 countries (the United Kingdom vs the United States) and for gender invariance (female vs male samples).

Fourth, we assessed the internal consistency (reliability) of the factorial structure by computing Cronbach α and McDonald ω coefficients for each dimension, as well as the corrected item–total correlation. Values of α and *ω* ≥0.80 (≥.70) indicate good (acceptable) internal consistency [49,50]. A good corrected item–total correlation is set at *r*≥0.30 [49,50].

Fifth, to test convergent validity, we conducted Pearson correlation analyses between participants’ total score on the PDWS, DFS, and DSS dimensions (as well as between the total score of these 3 scales).

Finally, we conducted moderation and mediation analyses, using the 2 smartphone-use variables (smartphone screen time and hours per day on a typical day using a smartphone for nonessential activities) as predictors; participants’ age, gender, and SES as moderators and mediators; and participants’ PDWS scores as the outcome variable.

The analyses were conducted using Jamovi statistics software (version 2.6 [51]) for descriptive, correlation, and moderation-mediation analyses and R statistical programming (package “Lavaan,” version 0.6‐19; Yves Rosseel; [52]) for CFA.

### Ethical Considerations

The study was conducted in accordance with the principles of the Declaration of Helsinki [53]. All participants provided digital informed consent for their survey contribution. That is, for the participants to be allowed to participate in the anonymous online survey, they were required to read a written consent form and sign it electronically by clicking a consent button. Participation was voluntary and restricted to those aged ≥18 years. All data were anonymously collected. Ethical approval (no KB 390/2022) was obtained from the Bioethics Committee of the Nicolaus Copernicus University functioning at Collegium Medicum in Bydgoszcz, Poland.

## Results

### Overview

Table 1 presents the descriptive statistics related to the participants’ sociodemographic characteristics and their smartphone use behavior.

**Table 1.** Descriptive statistics of sociodemographic characteristics and smartphone use behavior among participants from the United States and the United Kingdom (N=1854).

Variable	Scale	Min-Max	Frequency, mean (SD) or n (%)
US participants, n (%)	—^a^	—	934 (50.5)
UK participants, n (%)	—	—	920 (49.4)
Overall sociodemographic			
Age, mean (SD)	18-25	18‐25	22.4 (2.1)
Gender, n (%)	—	—	
Female			892 (48.1)
Male			872 (47.0)
Nonbinary			90 (4.9)
Sociodemographic for the US sample			
Age, mean (SD)	18‐25	18‐25	22.4 (2.08)
Gender, n (%)	—	—	
Female			444 (47.6)
Male			436 (46.7)
Nonbinary			53 (5.7)
Sexual orientation, n (%)	—	—	
Heterosexual			656 (70.4)
Homosexual			64 (6.9)
Bisexual			168 (17.9)
Other			33 (4.8)
Relationship status, n (%)	—	—	
Not in a relationship			395 (42.4)
In a relationship but not married			406 (43.5)
In a relationship and married			132 (14.2)
Ethnicity, n (%)	—	—	
White			510 (54.7)
Asian			80 (8.6)
Mixed			96 (10.3)
Black			214 (11.5)
Others			32 (3.4)
Education level^b^, mean (SD)	—	2‐25	15.4 (2.96)
Perceived socioeconomic status, n (%)	—	—	
Low			247 (26.5)
Intermediary			631 (67.7)
High			54 (5.8)
Sociodemographic for the UK sample			
Age	18‐25	18‐25	22.3 (2.11)
Gender, n (%)	—	—	
Female			448 (48.7)
Male			435 (47.3)
Nonbinary			37 (4.0)
Sexual orientation, n (%)	—	—	
Heterosexual			676 (73.5)
Homosexual			65 (7.1)
Bisexual			146 (15.9)
Other			45 (3.6)
Relationship status, n (%)	—	—	
Not in a relationship			448 (48.7)
In a relationship but not married			438 (47.7)
In a relationship and married			33 (3.6)
Ethnicity, n (%)	—	—	
White			622 (67.6)
Asian			127 (13.8)
Mixed			73 (7.9)
Black			84 (9.1)
Others			14 (1.5)
Education level^b^ (years of schooling), mean (SD)	—	2‐25	16.1 (2.64)
Perceived socioeconomic status, n (%)	—	—	
Low			266 (28.9)
Intermediary			612 (66.5)
High			42 (4.6)
Smartphone use behavior in the United States^b^, mean (SD)			
Smartphone screen time (hours per day)	—	1‐24	6.95 (3.87)
Smartphone time use for nonessential activities (hours per day)	1‐6	—	3.62 (1.35)
Smartphone use behavior in the United Kingdom^b^, mean (SD)			
Smartphone screen time (hours per day)	—	1‐24	6.13 (3.22)
Smartphone time use for nonessential activities (hours per day)	1‐6	1‐6	3.29 (1.19)
Most app used in the United States and in the United Kingdom, n (%)			
The 3 “first app most used”	—	—	
TikTok			482 (26.0)
Instagram			352 (19.0)
YouTube			111 (6.0)
The 5 “second app most used”	—	—	
Instagram			338 (18.2)
TikTok			237 (12.8)
YouTube			161 (8.7)
WhatsApp			121 (6.5)
Snapchat			91 (4.9)
PDWS^c^ total score^b^, mean (SD)			
United States	1‐5	—	3.49 (0.76)
United Kingdom	1‐5	—	3.38 (0.61)

a Not applicable.

b Regarding the scores related to these variables, all the differences between the United States and the United Kingdom were statistically significant: education level (*t*_1852_=5.25, *P*<.001, Cohen *d*=0.24); smartphone screen time (*t*_1852_=4.97, *P*<.001, *d*=0.27); smartphone time use for nonessential activities (*t*_1852_=5.57, P<.001, *d*=0.25); PDWS total score (*t*_1852_=3.33, *P*<.001, *d*=0.15).

c PDWS: Perceived Digital Well-Being Scale.

### Descriptive Statistics on the Participants’ Sociodemographic Variables

In the US sample, the percentage of women and men participants was very close (n=444, 47.6% and n=436, 46.7%, respectively), with very few identifying as nonbinary (n=53, 5.7%). The vast majority of participants reported being heterosexual (heterosexual: n=656, 70.4%; homosexual: n=64, 6.9%; bisexual: n=168, 17.9%; and other: n=33, 4.8%). Most of them were not married (not in a relationship: n=395, 42.4%; in a relationship but not married: n=406, 43.5%; and in a relationship and married: n=132, 14.2%). Most of them were ethnically White (White: n=510, 54.7%; Asian: n=80, 8.6%; Mixed: n=96, 10.3%; Black: n=214, 11.5%; and others: n=33, 1.8%). The average years of education was 15.4, and most participants self-reported belonging to the intermediate SES group (low: n=247, 26.5%; intermediate: n=631, 67.7%; and high: n=54, 5.8%).

In the UK sample, the percentage of women and men participants was very close (n=444, 48.7% and n=436, 47.3%, respectively), with very few identifying as nonbinary (n=53, 4.0%). The vast majority of participants reported being heterosexual (heterosexual: n=656, 73.5%; homosexual: n=64, 7.1%; bisexual: n=168, 15.9%; and other: n=33, 3.6%). Most were not married (not in a relationship: n=395, 48.7%; in a relationship but not married: n=406, 47.7%; in a relationship and married: n=132, 3.6%). The majority identified as White (White: n=510, 67.6%; Asian: n=80, 13.8%; Mixed: n=96, 7.9%; Black: n=214, 9.1%; and others: n=33, 1.5%). The average years of education was 16.1, and most participants self-reported belonging to the intermediate SES group (low: n=266, 28.9%; intermediate: n=612, 66.5%; and high: n=42, 4.6%).

### Descriptive and Inferential Statistics Related to Smartphone Use Behavior and Digital Well-being

As shown in Table 1, among both US and UK participants, TikTok (ByteDance), Instagram (Meta), and YouTube were respectively the first, second, and third most used apps. Comparatively, smartphone screen time and smartphone time use for nonessential activities were statistically higher in the US sample than in the UK sample (mean 6.95 vs mean 6.13; *t*_1852_=4.97; *P*<.001; *d*=0.27; and mean 3.62 vs mean 3.29; *t*_1852_=5.57; *P*<.001; *d*=0.25, respectively). Finally, the digital well-being total score was statistically higher among US participants compared with their UK counterparts (mean 3.49 vs mean 3.38; *t*_1852_=3.33; *P*<.001; *d*=0.15).

The quartile values of the PDWS total mean score were: first quartile=1‐3, second quartile=3‐3.47, third quartile=3.47‐3.88, fourth quartile=3.88‐5, respectively. Multimedia Appendix 1 shows the number of participants by quartile of the PDWS total mean score and by gender (women vs men). The women versus men distribution was statistically significant only for the first and fourth quartiles (lowest quartile: women: n=143, 39.94%; men: n=195, 54.46%; total: n=358, 100%; highest quartile: women: n=749, 52.52%; men: n=677, 47.47%; total: n=1426, 100%; *χ*^2^_136_=478.45; *P*<.001). This means that male participants are significantly more represented among the group of participants with higher DPWB scores.

### Descriptive Statistics of the PDWS Items

Table 2 displays the descriptive statistics related to the PDWS items. As shown in this table, all items had mean values above the middle of the 5-point scale. The skewness and the kurtosis values indicate a prevalence of a normally shaped distribution.

**Table 2.** Descriptive statistics related to the Perceived Digital Well-Being Scale (PDWS; 17 items grouped into 3 factors).

Factors and items	Scale	Mean (SD)	Skewness^a^	Kurtosis^b^
Factor 1: emotional domain (ED)	1‐5	3.39 (0.78)	−0.26	0.01
E1. Because of my smartphone use, I do fewer fun things, or because of my smartphone use, I do more fun things.	1‐5	3.35 (1.08)	−0.25	−0.68
E2. Because of my smartphone use, I feel more stressed, or because of my smartphone use, I feel more relaxed.	1‐5	3.23 (1.11)	−0.19	−0.75
E3. Because of my smartphone use, I feel bored, or because of my smartphone use, I feel entertained.	1‐5	3.86 (1.08)	−0.90	0.17
E4. Because of my smartphone use, I drop some leisure activities that I like, or because of my smartphone use, I engage in new leisure activities that I like.	1‐5	3.26 (1.10)	−0.15	−0.75
E5. Because of my smartphone use, I feel more upset, or because of my smartphone use, I feel calmer.	1‐5	3.32 (1.01)	−0.16	−0.43
E6. Because of my smartphone use, I feel sadder, or because of my smartphone use, I feel happier.	1‐5	3.38 (1.03)	−0.22	−0.50
E7. Because of my smartphone use, I feel my life is worse than most teens, or because of my smartphone use, I feel my life is better than most teens.	1‐5	3.31 (1.02)	−0.18	−0.31
Factor 2: social domain (SD)	1‐5	3.73 (0.83)	−0.65	0.28
S8. Because of my smartphone use, I feel more excluded from my friends, or because of my smartphone use, I feel closer to my friends.	1‐5	3.65 (1.10)	−0.55	-0.46
S9. Because of my smartphone use, I talk less with my friends, or because of my smartphone use, I talk more with my friends.	1‐5	3.93 (1.10)	−0.92	0.14
S10. Because of my smartphone use, I feel less informed about the lives of my friends, or because of my smartphone use, I feel more informed about the lives of my friends.	1‐5	4.05 (1.00)	−1.03	0.62
S11. Because of my smartphone use, I have fewer true friendships, or because of my smartphone use, I have more true friendships.	1‐5	3.42 (1.08)	−0.33	−0.45
S12. Because of my smartphone use, I feel less connected to my friends, or because of my smartphone use, I feel more connected to my friends.	1‐5	3.91 (1.02)	−0.91	0.26
S13. Because of my smartphone use, I spend less time with my friends, or because of my smartphone use, I spend more time with my friends.	1‐5	3.44 (1.10)	−0.32	−0.61
Factor 3: cognitive domain (CD**)**	1‐5	3.10 (0.88)	0.11	−0.24
C14. Because of my smartphone use, I do less of my daily tasks (eg, schoolwork, university work, and professional work), or because of my smartphone use, I do more of my daily tasks (eg, schoolwork, university work, and professional work).	1‐5	2.88 (1.15)	0.18	−0.76
C15. Because of my smartphone use, I have lower grades at school, at university, or low performance when doing an important work, or because of my smartphone use, I have high grades at school, at university, or high performance when doing an important work.	1‐5	3.22 (0.94)	0.01	0.10
C16. Because of my smartphone use, I am slower in my daily tasks (eg, schoolwork, university work, and professional work), or because of my smartphone use, I am faster in my daily tasks (eg, schoolwork, university work, and professional work).	1‐5	2.94 (1.17)	0.14	−0.85
C17. Because of my smartphone use, I feel I am a failure, or because of my smartphone use, I feel satisfied with myself.	1‐5	3.34 (1.08)	−0.23	−0.052

a A general guideline for skewness is that if the number is greater than +1 or lower than –1, this is an indication of a substantially skewed distribution.

b For kurtosis, a general guideline is that if the number is greater than +1, the distribution is too peaked.

### CFA on the PDWS Data

**Figure 1.**
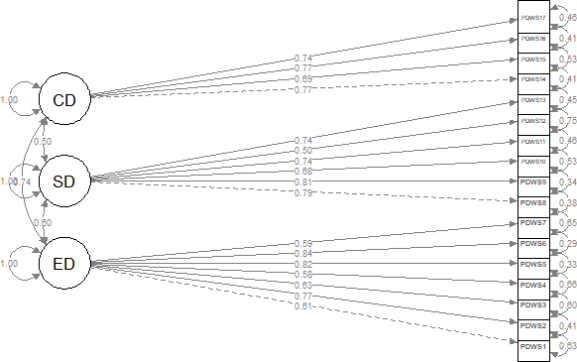
The Perceived Digital Well-Being Scale (PDWS) confirmatory factorial analysis’ path diagram with the standardized estimates. Confirmation factor analysis of the questionnaire. CD: cognitive domain; ED: emotional domain; SD: social domain.

The assumptions of adequacy (Kaiser–Meyer–Olkin=0.93) and sphericity (Bartlett test: *χ*^2^_136_==15,474.59; *P*<.001) were met [54]. Refer to Table 2 for all item labels. In Figure 1 the ellipses represent the latent variables (the 3 factors: emotional domain, social domain, and cognitive domain), and the rectangles represent the different items. The values on the arrows directed toward the ellipses indicate the variance of each latent factor (fixed to 1). The values on the arrows linking each of the 3 circles indicate the correlations between the latent variables. The values on the arrows linking each latent variable to the corresponding items represent the factor loadings (standardized estimates). The factor loadings indicate how well each item represents its unobservable construct (factor), with values ranging from 0 to 1. The values on top of each rectangle are the squares of the standardized factor loadings; they give the proportion of the explained variance (*R*^2^) in each item, which indicates how much of the variance in the item is explained by the unobserved construct. If a standardized factor loading value is greater than 0.70 or explains at least half of the variance (R^2^≥0.50) in the item, the corresponding item is important in explaining the unobserved construct it belongs to. Figure 1 displays the CFA path diagram. The overall model fit indices indicated a good fit between the model and the data (GFI=0.92, NFI=0.92, RFI=0.90, IFI=0.92, TLI=0.91, CFI=0.92, RMSEA=0.076, and SRMR=0.067). The factor loadings of the observed variables on the latent variable (refer to Figure 1 and Table S1 in Multimedia Appendix 2) ranged between 0.50 and 0.84 and were all significant (*P*<.001), indicating that each observed variable is a good indicator of the latent construct [47,48]. The variances of the observed variables (refer to Table S3 in the Multimedia Appendix 2) were also significant (*P*<.001), indicating variability in the measures [47,48]. The standardized estimates (refer to Tables S1-S4 in Multimedia Appendix 2) provide a way to compare the relative strengths of the relationships in a standardized form.

### Country (United States vs United Kingdom) Invariance Tests

To find out whether the PDWS factor structure was invariant across countries, a multigroup analysis was carried out. The configural invariance test showed an acceptable fit for the unconstrained model (GFI=0.90, NFI=0.91, RFI=0.89, IFI=0.92, TLI=0.91, CFI=0.92, RMSEA=0.073, and SRMR=0.066). The metric invariance test indicated that the meaning of the 3 modeled constructs (factors) did not statistically differ across the 2 countries (*χ*^2^_246_ change=17.90; *P*=.21).

### Gender (Women vs Men) Invariance Tests

To find out whether the PDWS factor structure was invariant across gender, a multigroup analysis was conducted. The configural invariance test showed an acceptable fit for the unconstrained model (GFI=0.90, NFI=0.90, RFI=0.89, IFI=0.92, TLI=0.91, CFI=0.92, RMSEA=0.074, and SRMR=0.066). However, the metric invariance test indicated that the meaning of the 3 modeled constructs (factors) significantly differed across groups (*χ*^2^_246_ change=200.91; *P*<.001).

### Internal Reliability

Table 3 shows the main results of the internal reliability tests conducted for each PDWS subscale.

The Cronbach α and the McDonald ω coefficients show that all PDWS dimensions had acceptable internal reliability (≥0.70). The corrected item–total correlation had *r*≥0.30.

**Table 3.** The Perceived Digital Well-Being Scale (PDWS) reliability statistics and item-total correlation.^a^

Factors and items	Subscale corrected item–total correlation^b^	Subscale McDonald ω if item deleted^c^
Factor 1: Emotional domain (ED); Cronbach *α*=0.86; McDonald *ω*=0.87		
E1. Because of my smartphone use, I do fewer fun things or because of my smartphone use, I do more fun things.	0.59	0.86
E2. Because of my smartphone use, I feel more stressed or because of my smartphone use, I feel more relaxed.	0.70	0.84
E3. Because of my smartphone use, I feel bored or because of my smartphone use, I feel entertained.	0.58	0.86
E4. Because of my smartphone use, I drop some leisure activities that I like or because of my smartphone use, I engage in new leisure activities that I like.	0.55	0.86
E5. Because of my smartphone use, I feel more upset or because of my smartphone use, I feel calmer.	0.73	0.83
E6. Because of my smartphone use, I feel sadder or because of my smartphone use, I feel happier.	0.76	0.83
E7. Because of my smartphone use, I feel my life is worse than most teens or because of my smartphone use, I feel my life is better than most teens.	0.53	0.86
Factor 2: social domain (SD); Cronbach *α*=0.87; McDonald *ω*=0.87		
S8. Because of my smartphone use, I feel more excluded from my friends or because of my smartphone use, I feel closer to my friends.	0.70	0.84
S9. Because of my smartphone use, I talk less with my friends or of my smartphone use, I talk more with my friends.	0.76	0.83
S10. Because of my smartphone use, I feel less informed about the lives of my friends or because of my smartphone use, I feel more informed about the lives of my friends.	0.63	0.85
S11. Because of my smartphone use, I have fewer true friendships or because of my smartphone use, I have more true friendships.	0.68	0.84
S12. Because of my smartphone use, I feel less connected to my friends or because of my smartphone use, I feel more connected to my friends.	0.53	0.87
S13. Because of my smartphone use, I spend less time with my friends or because of my smartphone use, I spend more time with my friends.	0.68	0.84
Factor 3: Cognitive domain (CD); Cronbach *α*=0.83; McDonald *ω*=0.83		
C14. Because of my smartphone use, I do less of my daily tasks (eg, schoolwork, university work, and professional work) or because of my smartphone use, I do more of my daily tasks (eg, schoolwork, university work, and professional work).	0.69	0.77
C15. Because of my smartphone use, I have lower grades at school, at university, or low performance when doing important work or because of my smartphone use, I have high grades at school, at university, or high performance when doing important work.	0.63	0.80
C16. Because of my smartphone use, I am slower in my daily tasks (eg, schoolwork, university work, and professional work) or because of my smartphone use, I am faster in my daily tasks (eg, schoolwork, university work, and professional work).	0.70	0.77
C17. Because of my smartphone use, I feel I am a failure or because of my smartphone use, I feel satisfied with myself.	0.61	0.81

a Regarding the Cronbach α, if the scale is an exploratory one, a good reliability is set at α>0.70. If the scale is an established one, a good reliability is set at α>0.80.

b A Item-Total Correlation: A good corrected item–total correlation is set at *r*>0.30.

c The McDonald ω if item deleted corresponds to values of McDonald ω if the relevant item is deleted. These metrics suggest that no item should be deleted.

### Correlations Between the PDWS Dimensions

All correlations were significant (*P*<.001). As shown in Figure 1, the strongest correlation was between the emotional domain and the cognitive domain.

### Convergent Validity

Table 4 presents the correlations between the 3 PDWS dimensions, the DFS dimensions, and the DSS dimensions, as well as between the total scores of these 3 scales. Almost all correlations were statistically significant, and most were strongly significant (*P*<.001). In addition, almost all correlations were in the expected direction. The exception regarding the direction of the relationships was between the PDWS dimensions and the DSS availability stress dimension. As shown in Table 4, the relationship between these factors was positive, which is counterintuitive. However, as hypothesized, the PDWS total score was negatively correlated with the DSS total score.

**Table 4.** Correlations between the Perceived Digital Well-Being Scale (PDWS), Digital Flourishing Scale (DFS), and multidimensional Digital Stress Scale (DSS).

Scales (factors)	PDWS			
	Emotional domain	Social domain	Cognitive domain	Total score
DFS factors, *r*				
Connectedness	0.47^a^	0.42^a^	0.40^a^	0.52^a^
Self-control	0.30^a^	0.32^a^	0.30^a^	0.36^a^
Civil participation	0.30^a^	0.20^a^	0.25^a^	0.30^a^
Positive social comparison	0.36^a^	0.34^a^	0.39^a^	0.43^a^
Authentic self-disclosure	0.49^a^	0.40^a^	0.47^a^	0.54^a^
DFS total score	0.54^a^	0.47^a^	0.51^a^	0.61^a^
DSS actors, *r*				
Connection overload	–0.06^b^	–0.07^b^	–0.01	–0.06^b^
Availability stress	0.08^b^	0.04^b^	0.08^a^	0.08^b^
Approval anxiety	–0.11^a^	–0.09^a^	–0.16^a^	–0.13^a^
Fear of missing out	–0.21^a^	–0.17^a^	–0.24^a^	–0.24^a^
Online vigilance	–0.07^b^	–0.03	–0.15^a^	–0.09^a^
DSS total score	–0.10^a^	–0.09^a^	–0.13^a^	–0.12^a^

a Correlation is significant at *P*≤.001.

b Correlation is significant at * P*<.05.

### Relationships Between PDWS Factors and Participants’ Sociodemographic and Smartphone Use Variables

Table 5 shows the correlation coefficients between the PDWS factors and the modeled sociodemographic and smartphone use variables.

**Table 5.** Associations between the 3 Perceived Digital Well-Being Scale (PDWS) factors, the participants’ sociodemographic characteristics, and smartphone use behavior.

Variable (factor)	PDWS			
	Emotional domain	Social domain	Cognitive domain	Total score
Sociodemographic				
Age, *r*	0.02	–0.00	0.05^a^	0.03
Gender^b^	—^c^	—	—	*F*_2,1852_=2.94; *P*=.05; η²_p_=0.003
Sexual orientation^d^	—	—	—	*F*_3,1851_=5.54; *P*<.001; η²_p_=0.009
Relationship status^e^	—	—	—	*F*_3,1851_=19.2; *P*<.001; η²_p_=0.020
Ethnicity^f^	—	—	—	*F*_4,1850_=9.65; *P*<.001; η²_p_=0.020
Education level (years of education), *r*	0.044	0.06^a^	0.03	0.06^a^
Perceived socioeconomic status, *r*	0.072^a^	0.04	0.10^g^	0.08^g^
Smartphone use behavior, *r*				
Smartphone screen time (hours per day)	0.09^g^	0.03	0.05^a^	0.07^a^
Smartphone time use for nonessential activities (hours per day)	0.09^g^	0.02	0.02	0.06^a^

a Correlation is significant at the *P*<.05.

b Gender x PDWS total score: women (mean 3.43, SD 0.68); men (mean 3.46, SD 0.70); nonbinary or other (mean 3.30, SD 0.70). The omnibus ANOVA showed no statistically significant difference, but pairwise comparison indicated a male vs nonbinary significant difference on the PDWS total score (*t*_1851_=2.42; *P*=.04; *d*=0.26).

c Not applicable.

d Sexual orientation x PDWS total score: heterosexual (mean 3.47, SD 0.69); homosexual (mean 3.39, SD 0.70); bisexual (mean 3.30, SD 0.67); other (mean 3.48, SD 0.69): The omnibus ANOVA showed a significant difference, and the pairwise comparison indicated that only heterosexual vs bisexual PDWS total scores were statistically significant (*t*_1851_=3.98; *P*<.001; *d*=0.25).

e Relationship status x PDWS total score: not in relationship (mean 3.35, SD 0.67; in a relationship but not married (mean 3.46, SD 0.69); in a relationship and married (mean 3.71, SD 0.74). The omnibus ANOVA showed a statistically significant difference, and all pairwise comparisons were significant (*t*_1851_=–3.23, *P*=.004, *d*=0.51; *t*_1851_=–5.99, *P*<.001; *t*_1851_=–4.14, *P*<.001, *d*=0.35; respectively).

f Ethnicity x PDWS total score: White (mean 3.75, SD 0.82); Asian (mean 3.59, SD 0.89); Mixed (mean 3.70, SD 0.79); Black (mean 3.74, SD 0.84); Others (mean 3.76, SD 1.03). The omnibus ANOVA showed a significant difference, and the pairwise comparison indicated that only the White vs Black, Asian vs Black, and Mixed vs Black PDWS total scores were statistically significant (*t*_1849_=–5.76, *P*<.001, *d*= 0.37; *t*_1849_=–5.13, *P*<.001, *d*=0.46; *t*_1849_=–3.75, *P*=.002, *d*=.36; respectively).

g Correlation is significant at *P*≤.001.

Participants’ age was significantly and positively correlated with the PDWS cognitive domain, indicating that older participants in our sample were more likely to experience higher levels of digital cognitive well-being compared to younger participants. The PDWS total score among male participants was statistically higher than among nonbinary participants. Overall, there was no significant difference in the scores between female and male participants. However, when comparing the lowest and highest quartiles, as reported in the “Descriptive results” subsection, female participants had significantly lower digital well-being than male participants. The PDWS total score for heterosexual participants was statistically higher than for bisexual participants, whereas there was no significant difference between the scores of the heterosexual and homosexual participants.

Participants’ education level was significantly and positively correlated with the PDWS social domain, indicating that the highly educated participants were more likely to experience social well-being compared to those with lower levels of education. Overall, participants’ self-reported SES was significantly and positively correlated with their digital well-being; this was particularly true for the emotional and cognitive domains. Curiously, participants’ smartphone screen time and self-reported time spent on nonessential smartphone activities were both significantly and positively correlated with the overall PDWS score; this was especially true regarding the emotional domain.

### Moderation and Mediation Effects

Gender significantly moderated the relationship between nonessential smartphone use and participants’ PDWS scores, as indicated by a beta coefficient (*b*=0.0489, 95% CI 0.005-0.092; *P*=.03). Similarly, SES emerged as a significant moderator in the association between smartphone screen time and PDWS scores (*b*=0.0205, CI 0.002-0.038; *P*=.03). In contrast, no significant moderating effect of age was observed. Moreover, gender, age, and SES did not serve as significant mediators in the relationship between smartphone use and PDWS scores.

## Discussion

### Principal Findings

Digital well-being may serve as a critical indicator of the relationship between digital connectivity and individuals’ mental health or overall well-being [17,25,27,28]. Accordingly, the ability to assess digital well-being holds potential benefits for both research and practical interventions. This study aimed to validate the English version for both adolescents and adults of the PDWBA Scale and to examine the associations between its 3 dimensions and a range of sociodemographic and behavioral variables.

### Factor Structure and Psychometric Properties

First, the results of this study support the robustness of the 3-factor, 17-item structure of the English adolescent-adult version of the PDWBA (named, in this study, PDWS), indicating its suitability for assessing digital well-being in both the US and the UK contexts. CFA revealed that the model demonstrated a good fit across both national samples, aligning with the theoretical framework of the original scale (the PDWBA [17]). Fit indices were all in the good-excellent range. These findings provide empirical support for the generalizability of the PDWS among young adult populations in the United States and the United Kingdom.

Second, internal consistency analyses showed satisfactory reliability across all 3 dimensions of the PDWS (Cronbach α≥0.83), consistent with previous validations of the PDWBA [17]. Furthermore, the scale demonstrated strong item discrimination, with corrected item–total correlations of ≥0.53 in both samples. Internal reliability was also robust, with all factor loadings above 0.50, suggesting that the PDWS adequately captures the core dimensions of digital well-being among the target population.

Third, the convergent validity of the PDWS was supported by its statistically significant correlations with established measures of digital flourishing (DFS [13,14]) and digital stress (DSS [10]). All 3 PDWS dimensions exhibited strong, positive correlations with the 5 DFS dimensions, suggesting that higher digital well-being, as measured by the PDWS, is associated with greater experiences of digital flourishing. Digital flourishing encompasses positive user experiences and perceptions of computer-mediated interactions and is grounded in the broader positive psychology framework of flourishing, conceptualized as both “feeling well” and “doing well” [55,56]. Flourishing’s affective component—“feeling well”—aligns with subjective well-being [19], whereas the behavioral and functional components—“doing well”—align with eudaimonic well-being [20], encompassing dimensions such as purpose, self-actualization, connectedness, and mastery [57,58]. These findings suggest that digital well-being contributes not only to emotional well-being but also to functional flourishing in both online and offline contexts. Conversely, digital stress—defined as the psychological burden associated with social media and online service use—has been linked to diminished mental health, particularly among adolescents and young adults [10,42,43,59]. Consistent with this body of research, the PDWS was significantly and negatively associated with all DSS dimensions—approval anxiety, fear of missing out, connection overload, and online vigilance, exception of one. Notably, all PDWS dimensions were positively correlated with the DSS availability stress subscale. This subscale reflects the perceived pressure and social obligation to remain constantly accessible via digital platforms, shaped by internalized expectations from peers [10]. This unexpected finding warrants further investigation, as it may indicate that some aspects of digital well-being are accompanied by heightened awareness of social availability demands. Future research should explore this relationship across different demographic and behavioral subgroups, perhaps through cluster analysis, to determine whether divergent patterns exist within specific populations and how these patterns relate to digital behavior and sociodemographic variables. Furthermore, the wide distribution of PDWS scores across the 4 quartiles supports the need to conduct such studies.

Fourth, measurement invariance testing confirmed that the PDWS captures the same underlying construct of digital well-being across national contexts (United States vs United Kingdom), supporting its cross-cultural applicability. However, the results indicated a lack of measurement invariance across gender, particularly in factor loadings and item intercepts. This suggests potential bias in digital well-being assessments between male and female participants. Previous studies [60-62] have reported gender-related differences in digital experiences, noting that women are more likely to engage in social media for relational purposes and exhibit higher tendencies toward social comparison. Furthermore, females tend to participate more in online social communities, which may increase exposure to the negative consequences of digital connectivity, including stigma and cyberbullying [63,64]. These findings may partly explain the observed gender-related disparities in PDWS outcomes and underscore the importance of considering gender-specific factors when assessing digital well-being.

In summary, this study provides strong evidence for the validity and reliability of the PDWS as a tool for assessing digital well-being in English-speaking adult populations. Its significant associations with digital flourishing and digital stress further support its criterion validity, although gender-specific differences and the unique role of availability stress warrant further exploration.

### Associations With Sociodemographic and Smartphone Use Behavior

First, the findings of this study indicate that older participants reported significantly higher levels of digital well-being compared to younger individuals, particularly in the domain of digital cognitive well-being. This pattern is consistent with previous research by Rosič et al [17]. A possible explanation for this age-related difference may lie in the development of reflective smartphone use disengagement, which tends to increase as individuals progress through adolescence into adulthood and further [9,65,66]. Based on the reflective-impulsive model [67], reflective smartphone use disengagement refers to the deliberate regulation of smartphone and social media use through conscious control mechanisms. These findings suggest that older individuals may be more adept at exercising self-regulation in digital environments, thereby enhancing their overall digital well-being. In terms of gender identity, male participants reported significantly higher digital well-being compared to other groups, while nonbinary individuals reported the lowest levels. Although Rosič et al [17] found that women perceived their emotional regulation as lower than men, their study did not examine digital well-being among nonbinary individuals. These findings may reflect broader patterns of digital engagement and vulnerability; for example, women are more frequently exposed to the negative psychological impacts of problematic smartphone and social media use [60,68]. In addition, individuals from gender and sexual minority groups—such as nonbinary and same-sex oriented participants—may encounter greater levels of online stigmatization and cyberbullying, which can adversely affect their digital well-being [63,64]. In this study, participants with heterosexual orientation reported significantly higher digital well-being than participants with bisexual orientation, further reinforcing the potential role of social identity in shaping digital experiences.

Second, higher levels of education were associated with significantly greater social digital well-being and cognitive digital well-being. Similarly, SES was positively correlated with digital well-being, particularly in the emotional and cognitive domains. These results align with the findings of Priyanka [27], who reported a positive relationship between social capital and digital well-being. It is plausible that individuals with higher educational attainment are more likely to develop effective strategies for reflective disengagement from digital technologies [65,66]. Moreover, individuals from higher SES backgrounds may have broader access to offline recreational and social opportunities, reducing their reliance on digital platforms and mitigating the negative effects of excessive digital engagement.

Third, participants’ smartphone screen time and usage of smartphones for nonessential activities were both significantly and positively correlated with overall PDWS scores—a finding contrary to initial expectations. One possible explanation is that, in the present sample, greater screen time may not necessarily reflect problematic use but could instead be associated with activities that enhance subjective well-being, such as meaningful social interaction, entertainment, or educational content. This highlights the need for future research to disaggregate types of digital activity and examine their distinct dynamic associations with digital well-being. Subgroup analyses could further clarify whether specific patterns of use are differentially associated with well-being across various demographic and behavioral profiles.

Fourth, the present findings provide nuanced insights into the psychosocial correlates of smartphone use by demonstrating that both gender and SES significantly moderate the relationship between smartphone use and psychological distress, as measured by PDWS. Specifically, nonessential smartphone use was more strongly associated with PDWS scores in one gender group than another, as indicated by the significant interaction effect. This aligns with previous research suggesting that women may experience higher levels of psychological distress linked to digital overuse, potentially due to greater social comparison, emotional engagement with online content, or differential patterns of use [69]. Similarly, SES significantly moderated the association between overall screen time and PDWS scores, suggesting that individuals from lower SES backgrounds may be more vulnerable to the psychological impacts of excessive smartphone use. This may reflect limited access to coping resources, higher exposure to stressors, or a greater reliance on smartphones for escapism and entertainment in lower-income populations. These findings support the “digital stress divide” hypothesis, which posits that the psychological consequences of digital media are not evenly distributed across social groups [70]. Interestingly, age did not significantly moderate the relationship, suggesting that the psychological effects of smartphone use are relatively stable across age groups within the sample. This finding challenges the assumption that younger users are uniquely vulnerable and encourages further exploration into qualitative differences in how smartphone use affects mental health across the lifespan. In addition, the lack of significant mediation effects indicates that gender, age, and SES do not act as explanatory pathways through which smartphone use influences PDWS scores. Instead, their role appears to be more conditional—shaping the strength or direction of the effect rather than transmitting it. This distinction is critical, suggesting that interventions may need to be tailored based on demographic vulnerability rather than assuming a uniform psychological mechanism. These findings have practical implications for mental health interventions and digital wellness programs. For instance, gender-sensitive and socioeconomically inclusive strategies may be more effective in mitigating the negative effects of smartphone use. Future research should further investigate the underlying mechanisms—such as emotion regulation, digital literacy, or social support—that may explain why certain groups are more affected by smartphone overuse. In addition, it would be valuable to explore other potential moderators or mediators, including personality traits (eg, neuroticism), psychological resilience, and specific smartphone activities (eg, doom scrolling vs productivity apps), to build a more comprehensive model of digital distress/well-being.

Finally, the finding that the digital well-being total score was statistically higher among US participants when compared with the UK counterparts suggests a significant national-level difference in how individuals perceive or experience digital well-being. Interpreted within an academic framework, this result implies that participants from the United States reported more favorable outcomes or engagement with digital well-being practices or perceptions than those from the United Kingdom, with the difference being supported by statistical significance. Several factors may contribute to this disparity. These observed national differences between the United States and the United Kingdom suggest that digital well-being is influenced not only by individual behavior but also by broader cultural and policy environments [71]. The higher PDWS scores among US participants may reflect more prominent public discourse and initiatives around digital wellness, distinct norms of technology use, or cultural differences in how mental health and digital practices are understood and reported. These findings indicate that interventions to promote digital well-being should be culturally tailored, accounting for sociotechnical infrastructures, normative expectations surrounding technology, and the dynamic interplay between individuals and their digital environments [25].

In relation to previous research, this study confirms the robustness of the PDWS [17] while extending its applicability beyond adolescence to young adults in 2 cultural contexts. The present findings show that the 3-factor model holds in US and UK samples, supporting its generalizability. The observed links with digital flourishing and stress align with previous work [27,33,72]. The positive association with availability stress introduces a novel insight, suggesting that perceived accessibility may not always undermine well-being. Furthermore, by documenting differences not only across gender but also among nonbinary and sexual minority participants, the study expands the scope of digital well-being research to populations often overlooked. Overall, this study strengthens the evidence base for the PDWS and contributes new perspectives on demographic and cultural variations in digital well-being.

### Limitations

This study has several limitations that should be acknowledged. First, it relied exclusively on self-reported measures, which may be subject to social desirability bias, recall errors, and other self-reporting inaccuracies. Second, its cross-sectional design limits the ability to infer causality between digital well-being, flourishing, stress, and patterns of smartphone use. Third, although the sample size was large and included participants from both the United States and the United Kingdom, the recruitment method may not ensure full representativeness of young adult populations in these countries, potentially limiting generalizability.

### Future Research

Future research may benefit from a mixed methods approach to better contextualize these quantitative findings, examining how demographic, socioeconomic, and policy-level factors intersect to influence digital well-being across different populations. Thus, future research should adopt quantitative, longitudinal, international, and ecological designs [73] capable of capturing the dynamic interactions that shape digital well-being, including individual, social, and technological interface factors [25]. In addition, qualitative studies are warranted to explore the subjective dimensions of human–digital interactions at cognitive, emotional, social, and behavioral levels. The involvement of people with lived experience [74] is also crucial to refining assessments, conceptual frameworks, and intervention models. Collectively, such studies are essential for developing interventions tailored to the diverse needs of individuals experiencing difficulties in their digital well-being.

### Conclusion

The PDWS may serve as a useful tool to identify individuals at risk of difficulties linked to digital connectivity and to highlight potential gender- and identity-related disparities. It could also provide guidance for educators in promoting digital literacy, support clinicians in developing targeted interventions, and inform policymakers in shaping evidence-based programs. In this way, the scale may help facilitate new avenues for fostering healthier digital ecosystems at both individual and societal levels.

## Supplementary material

10.2196/78334 Multimedia Appendix 1 Number of participants (female vs male) by quartile of the Perceived Digital Well-Being Scale (PDWS) total mean score.

10.2196/78334 Multimedia Appendix 2 Additional material, including the latent variables parameters estimate, covariances estimate, variance estimate, and parameters estimate.

## Abbreviation

CFA: confirmatory factorial analysis
CFI: comparative fit index
DFS: Digital Flourishing Scale
DSS: Digital Stress Scale
DWQ: Digital Well-Being Questionnaire
GFI: goodness-of-fit index
IFI: incremental fit index
NFI: normed fit index
PDWBA: Perceived Digital Well-Being in Adolescence Scale
PDWS: Perceived Digital Well-Being Scale
RFI: relative fit index
RMSEA: root mean square error of approximation
SES: socioeconomic status
SRMR: standardized root mean square residual
TLI: Tucker-Lewis index
