# Supplementary figures and images for: Perceived Digital Well-Being Scale in the United States and United Kingdom: Psychometric Validation Study
Source: JMIR Ment Health. 2025 Oct 30;12:e78334. doi: 10.2196/78334 (PMC12574938; doi:10.2196/78334)

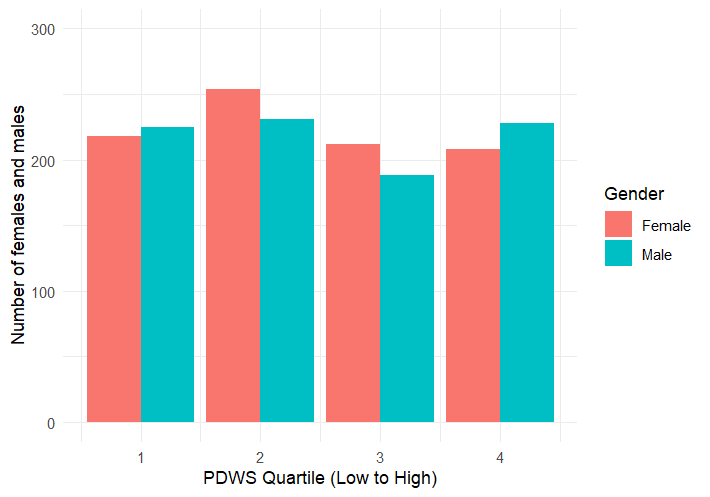

Supplement: Multimedia Appendix 1 [file mental-v12-e78334-s001.png]
