# Supplementary material for: Perceived Digital Well-Being Scale in the United States and United Kingdom: Psychometric Validation Study
Source: JMIR Ment Health. 2025 Oct 30;12:e78334. doi: 10.2196/78334 (PMC12574938; doi:10.2196/78334)
Supplement: Multimedia Appendix 2 [file mental-v12-e78334-s002.docx]

**Table S1.** Latent variables parameters estimate.

| Factors | Items | Estimate | SE | *z* value | *P* (>\|z\|) | Std.lv | Std.all |
| --- | --- | --- | --- | --- | --- | --- | --- |
| ED | PDWS1 | 1 |  |  |  | 0.66 | 0.61 |
| ED | PDWS2 | 1.29 | 0.04 | 26.30 | <0.001 | 0.85 | 0.77 |
| ED | PDWS3 | 1.03 | 0.04 | 22.74 | <0.001 | 0.68 | 0.63 |
| ED | PDWS4 | 0.97 | 0.04 | 21.35 | <0.001 | 0.64 | 0.58 |
| ED | PDWS5 | 1.24 | 0.04 | 27.38 | <0.001 | 0.82 | 0.81 |
| ED | PDWS6 | 1.31 | 0.04 | 27.93 | <0.001 | 0.87 | 0.84 |
| ED | PDWS7 | 0.90 | 0.04 | 21.53 | <0.001 | 0.59 | 0.58 |
| SD | PDWS8 | 1 |  |  |  | 0.86 | 0.78 |
| SD | PDWS9 | 1.03 | 0.02 | 36.22 | <0.001 | 0.89 | 0.81 |
| SD | PDWS10 | 0.79 | 0.02 | 29.74 | <0.001 | 0.68 | 0.68 |
| SD | PDWS11 | 0.92 | 0.02 | 32.40 | <0.001 | 0.79 | 0.73 |
| SD | PDWS12 | 0.52 | 0.02 | 21.05 | <0.001 | 0.44 | 0.50 |
| SD | PDWS13 | 0.94 | 0.02 | 32.72 | <0.001 | 0.81 | 0.74 |
| CD | PDWS14 | 1 |  |  |  | 0.88 | 0.76 |
| CD | PDWS15 | 0.73 | 0.02 | 28.32 | <0.001 | 0.65 | 0.68 |
| CD | PDWS16 | 1.02 | 0.03 | 31.83 | <0.001 | 0.90 | 0.77 |
| CD | PDWS17 | 0.90 | 0.03 | 30.48 | <0.001 | 0.79 | 0.73 |

ED = emotional domain; SD = social domain; CD = cognitive domain; P = *P* value; Std.lv & Std.all = standard estimates. PDWS = Perceived Digital Wellbeing Scale. **Note**. In the context of CFA, the Std.all is the most relevant estimate.

**Table S2.** Covariances estimate.

| Factors | Estimate | SE | z-value | P (>\|z\|) | Std.lv | Std.all |
| --- | --- | --- | --- | --- | --- | --- |
| ED-SD | 0.34 | 0.02 | 16.37 | <0.001 | 0.59 | 0.59 |
| ED-CD | 0.42 | 0.02 | 17.75 | <0.001 | 0.73 | 0.73 |
| SD-CD | 0.38 | 0.02 | 15.48 | <0.001 | 0.49 | 0.49 |

ED = emotional domain; SD = social domain; CD = cognitive domain. PDWS = Perceived Digital Wellbeing Scale. **Note.** In the context of CFA, the Std.all is the most relevant estimate.

**Table S3.** Variance estimate.

| Items | Estimate | SE | *z* value | *P* (>\|z\|) | Std.lv | Std.all |
| --- | --- | --- | --- | --- | --- | --- |
| PDWS1 | 0.73 | 0.02 | 28.51 | <0.001 | 0.73 | 0.62 |
| PDWS2 | 0.50 | 0.02 | 25.66 | <0.001 | 0.50 | 0.40 |
| PDWS3 | 0.70 | 0.02 | 28.29 | <0.001 | 0.70 | 0.60 |
| PDWS4 | 0.80 | 0.02 | 28.77 | <0.001 | 0.80 | 0.66 |
| PDWS5 | 0.33 | 0.01 | 23.77 | <0.001 | 0.33 | 0.33 |
| PDWS6 | 0.30 | 0.01 | 22.29 | <0.001 | 0.30 | 0.28 |
| PDWS7 | 0.67 | 0.02 | 28.72 | <0.001 | 0.67 | 0.65 |
| PDWS8 | 0.46 | 0.01 | 23.83 | <0.001 | 0.46 | 0.38 |
| PDWS9 | 0.41 | 0.01 | 22.60 | <0.001 | 0.41 | 0.34 |
| PDWS10 | 0.53 | 0.02 | 26.92 | <0.001 | 0.53 | 0.53 |
| PDWS11 | 0.53 | 0.02 | 25.67 | <0.001 | 0.53 | 0.45 |
| PDWS12 | 0.60 | 0.02 | 29.11 | <0.001 | 0.60 | 0.75 |
| PDWS13 | 0.54 | 0.02 | 25.48 | <0.001 | 0.54 | 0.45 |
| PDWS14 | 0.54 | 0.02 | 22.91 | <0.001 | 0.54 | 0.41 |
| PDWS15 | 0.47 | 0.01 | 25.80 | <0.001 | 0.47 | 0.52 |
| PDWS16 | 0.55 | 0.02 | 22.73 | <0.001 | 0.55 | 0.40 |
| PDWS17 | 0.53 | 0.02 | 24.19 | <0.001 | 0.53 | 0.45 |
| ED | 0.43 | 0.03 | 13.95 | <0.001 | 1 | 1 |
| SD | 0.74 | 0.03 | 19.35 | <0.001 | 1 | 1 |
| CD | 0.77 | 0.04 | 18.29 | <0.001 | 1 | 1 |

ED = emotional domain; SD = social domain; CD = cognitive domain; *P* = *P* value; Std.lv & Std.all = standard estimates. PDWS = Perceived Digital Wellbeing Scale. **Note.** In the context of CFA, the Std.all is the most relevant estimate.

**Table S4.** Parameters estimate.

| Factors | Items | Estimate | SD | z | p-value | CI (lower) | CI (upper) | std.lv | std.all |
| --- | --- | --- | --- | --- | --- | --- | --- | --- | --- |
| ED | PDWS1 | 1 | 0 |  |  | 1 | 1 | 0.65 | 0.61 |
| ED | PDWS2 | 1.29 | 0.04 | 26.30 | <0.001 | 1.19 | 1.38 | 0.85 | 0.76 |
| ED | PDWS3 | 1.03 | 0.04 | 22.74 | <0.001 | 0.94 | 1.12 | 0.68 | 0.63 |
| ED | PDWS4 | 0.97 | 0.04 | 21.35 | <0.001 | 0.88 | 1.05 | 0.64 | 0.58 |
| ED | PDWS5 | 1.24 | 0.04 | 27.38 | <0.001 | 1.15 | 1.33 | 0.82 | 0.81 |
| ED | PDWS6 | 1.31 | 0.04 | 27.93 | <0.001 | 1.22 | 1.41 | 0.87 | 0.84 |
| ED | PDWS7 | 0.90 | 0.04 | 21.53 | <0.001 | 0.82 | 0.98 | 0.59 | 0.58 |
| SD | PDWS8 | 1 | 0 |  |  | 1 | 1 | 0.86 | 0.78 |
| SD | PDWS9 | 1.03 | 0.02 | 36.22 | <0.001 | 0.97 | 1.09 | 0.89 | 0.81 |
| SD | PDWS10 | 0.78 | 0.02 | 29.73 | <0.001 | 0.73 | 0.84 | 0.68 | 0.68 |
| SD | PDWS11 | 0.91 | 0.02 | 32.40 | <0.001 | 0.86 | 0.97 | 0.79 | 0.73 |
| SD | PDWS12 | 0.51 | 0.02 | 21.05 | <0.001 | 0.47 | 0.56 | 0.44 | 0.50 |
| SD | PDWS13 | 0.94 | 0.02 | 32.72 | <0.001 | 0.89 | 1.00 | 0.81 | 0.74 |
| CD | PDWS14 | 1 | 0 |  |  | 1 | 1 | 0.88 | 0.76 |
| CD | PDWS15 | 0.73 | 0.02 | 28.32 | <0.001 | 0.68 | 0.78 | 0.65 | 0.68 |
| CD | PDWS16 | 1.02 | 0.03 | 31.83 | <0.001 | 0.96 | 1.08 | 0.90 | 0.77 |
| CD | PDWS17 | 0.90 | 0.02 | 30.48 | <0.001 | 0.84 | 0.96 | 0.79 | 0.73 |

ED = emotional domain; SD = social domain; CD = cognitive domain; SD = standard deviation; CI = confidence interval; Std.Err = standard error; P = p-value; Std.lv & Std.all = standard estimates. PDWS = Perceived Digital Wellbeing Scale. **Note.** In the context of CFA, the Std.all is the most relevant estimate.
